# Microbial Glycoside Hydrolases in the First Year of Life: An Analysis Review on Their Presence and Importance in Infant Gut

**DOI:** 10.3389/fmicb.2021.631282

**Published:** 2021-05-28

**Authors:** Athanasia Ioannou, Jan Knol, Clara Belzer

**Affiliations:** ^1^Laboratory of Microbiology, Wageningen University & Research, Wageningen, Netherlands; ^2^Danone Nutricia Research, Utrecht, Netherlands

**Keywords:** gut microbiota, human milk oligosaccharides, functional metagenomics, carbohydrate active enzymes, glycoside hydrolases, microbial communities

## Abstract

The first year of life is a crucial period during which the composition and functionality of the gut microbiota develop to stabilize and resemble that of adults. Throughout this process, the gut microbiota has been found to contribute to the maturation of the immune system, in gastrointestinal physiology, in cognitive advancement and in metabolic regulation. Breastfeeding, the “golden standard of infant nutrition,” is a cornerstone during this period, not only for its direct effect but also due to its indirect effect through the modulation of gut microbiota. Human milk is known to contain indigestible carbohydrates, termed human milk oligosaccharides (HMOs), that are utilized by intestinal microorganisms. Bacteria that degrade HMOs like *Bifidobacterium longum* subsp. *infantis*, *Bifidobacterium bifidum*, and *Bifidobacterium breve* dominate the infant gut microbiota during breastfeeding. A number of carbohydrate active enzymes have been found and identified in the infant gut, thus supporting the hypothesis that these bacteria are able to degrade HMOs. It is suggested that via resource-sharing and cross-feeding, the initial utilization of HMOs drives the interplay within the intestinal microbial communities. This is of pronounced importance since these communities promote healthy development and some of their species also persist in the adult microbiome. The emerging production and accessibility to metagenomic data make it increasingly possible to unravel the metabolic capacity of entire ecosystems. Such insights can increase understanding of how the gut microbiota in infants is assembled and makes it a possible target to support healthy growth. In this manuscript, we discuss the co-occurrence and function of carbohydrate active enzymes relevant to HMO utilization in the first year of life, based on publicly available metagenomic data. We compare the enzyme profiles of breastfed children throughout the first year of life to those of formula-fed infants.

## Introduction

The relationship between humans and the gut microbiota starts directly after birth and continues throughout life. The newborn gut is inoculated at birth with microorganisms that will be its first inhabitants. Through ecological succession, the infant gut gets enriched with microorganisms, and after the first year of life begins to reach a certain compositional stability (Lozupone et al., 2012). Several factors have been shown to influence the development of the gut microbiota composition in infants. The mode of birth, the gestation age, the type of feeding, the use of antibiotics, the environment and the mother’s secretor status are major components of this equation (Harmsen et al., 2000; Marques et al., 2010; Fouhy et al., 2012; Azad et al., 2013; Lewis et al., 2015). Even though the gut microbiota establishes a stable community with similarities to that of an adult roughly after the first 12–36 months (Lozupone et al., 2012; Bäckhed et al., 2015; Bokulich et al., 2016; Stewart et al., 2018), the infant microbiota preceding this period can affect lifelong health (Cox et al., 2014; O’Mahony et al., 2014; Serino et al., 2017; Carlson et al., 2018). The microbiota-mediated health effect in children is highly driven by the feeding in early life and sets breastfeeding as an important steering wheel of this process. The breast-milk derived palette of the infant gut microbiota has been associated, among others, with limited tendency to develop obesity (Luoto et al., 2011; Forbes et al., 2018), atopy (Fujimura et al., 2016) as well as various immunomodulatory factors (Schwartz et al., 2012; Fujimura et al., 2016). Even though bold associations are still controversial, several studies have found links between breastfeeding and lower occurrence of diseases like asthma (Silvers et al., 2012; Ahmadizar et al., 2017; Xue et al., 2019; Harvey et al., 2020) and eczema (Chiu et al., 2016; Elbert et al., 2017; Flohr et al., 2018), limited tendency for obesity (Yamakawa et al., 2013; Yan et al., 2014; Bider-Canfield et al., 2017; Modrek et al., 2017) and better cognition development (Angelsen et al., 2001; Boucher et al., 2017; Lenehan et al., 2020). Research endeavors focus on human milk because the “golden standard” of feed could also be the “golden ticket” for improving alternative infant nutrition.

Human milk derives its nutritious value from its complex composition. It is a conglomeration of energy storing macromolecules, namely proteins, carbohydrates and fat, and bioactive compounds, such as immune cells, hormones, antimicrobials, vitamins, and glycans of various sizes (Ballard and Morrow, 2013). The glycans are commonly termed as human milk oligosaccharides (HMOs). The bonds holding the structure of HMOs are not degraded in the upper gastrointestinal tract, thus they are indigestible carbohydrates. More than 95% of the HMOs reach the infant’s gut undigested (Engfer et al., 2000; Gnoth et al., 2000). There they can be utilized by certain bacteria that can degrade them, and quickly after the beginning of lactation, the gut microbiota is dominated by taxa belonging to the phylum Firmicutes, Bacteroidetes, Actinobacteria and Proteobacteria (Stark and Lee, 1982; Penders et al., 2006; Adlerberth and Wold, 2009; Albrecht et al., 2013; Bäckhed et al., 2015). Bifidobacteria, especially the *Bifidobacterium bifidum*, *Bifidobacterium breve*, and *Bifidobacterium longum* subsp. *infantis*, have proven to be ample HMO-degraders, and a number of their enzymes have been isolated (Møller et al., 2001; Wada et al., 2008; Yoshida et al., 2012). Genomic-based analysis and *in vitro* experiments have shown that the enzymatic repertoire related to HMO degradation is probably species or even strain-specific (Pokusaeva et al., 2011; James et al., 2016; Sakanaka et al., 2020). Since the complete dismantling of HMOs dictates enzymes for transportation, degradation, and utilization a certain collaboration is suggested. Indeed, different strains of the same species have been identified as being part of microbial communities, intra- and inter- individually (Asnicar et al., 2017; Lawson et al., 2019), thus adding to the known collaborative substrate utilization between bifidobacteria (Milani et al., 2015). Moreover, other species such as *Bacteroides* spp., *Ruminococcus gnavus*, *Lactobacillus* spp., *Akkermansia muciniphila*, *Clostridium* spp., and *Escherichia coli* have also been found to possess the ability to degrade certain HMOs or parts of them in mono- and cocultures (Marcobal et al., 2011; Yu et al., 2013; Thongaram et al., 2017; Kostopoulos et al., 2020; Wu et al., 2020; Salli et al., 2021). Metabolic products are exchanged between bacteria via cross-feeding, creating a microbial network that collaboratively thrives in the presence of human milk carbohydrates (Bunesova et al., 2016; Schwab et al., 2017).

However, the gut microbiota composition of formula-fed infants has been found to be more diverse with higher prevalence and/or abundance of bacteria such as *Clostridium difficile*, *E. coli*, *Veillonella* spp., *Clostridioides* (formerly *Clostridium*), *Streptococcus* spp., *Enterococcus* spp., and adult-associated bifidobacteria (Penders et al., 2006; Fallani et al., 2010; Azad et al., 2013; Gomez-Llorente et al., 2013; Bäckhed et al., 2015; Ma et al., 2020). However, for taxa such as those from the genus *Bacteroides* and *Lactobacillus* there is not a clear consensus in literature (Fallani et al., 2010; Gomez-Llorente et al., 2013; Bäckhed et al., 2015; Ma et al., 2020). Introduction of solid food is also a factor that has been shown to shift the microbiome toward a more adult-like state (Bergström et al., 2014; Bäckhed et al., 2015) and was recently associated with taxa such as *A. muciniphila*, *Bacteroides* spp., *Erwinia* spp., *Streptococcus* spp., and *Veillonella* spp. (Differding et al., 2020). These findings demonstrate that human milk can be a major driver for the gut microbiota during this critical window. It is suggested that these profiles of infants, receiving or not receiving, human milk derive from the metabolic pressure applied by the presence and absence of HMOs, respectively. Therefore, it is of interest to explore the Carbohydrate Active Enzymes (CAZymes) profiles of the gut microbiota in milk-fed infants that are relevant to HMO-degradation. In this analysis review we focus on glycoside hydrolases related to HMO-degradation within the first year of life and current advances concerning their presence and importance. We compared these profiles to that of children who were formula fed. To assist our objective, we employed publicly available Metagenome Assembled Genomes (MAGs; Nayfach et al., 2019) based on metagenomic data from infants up to 12 months old (Bäckhed et al., 2015) with various feeding backgrounds.

## Energy Storing Glycans of Human Milk and Alternative Infant Feeding

**FIGURE 1:**
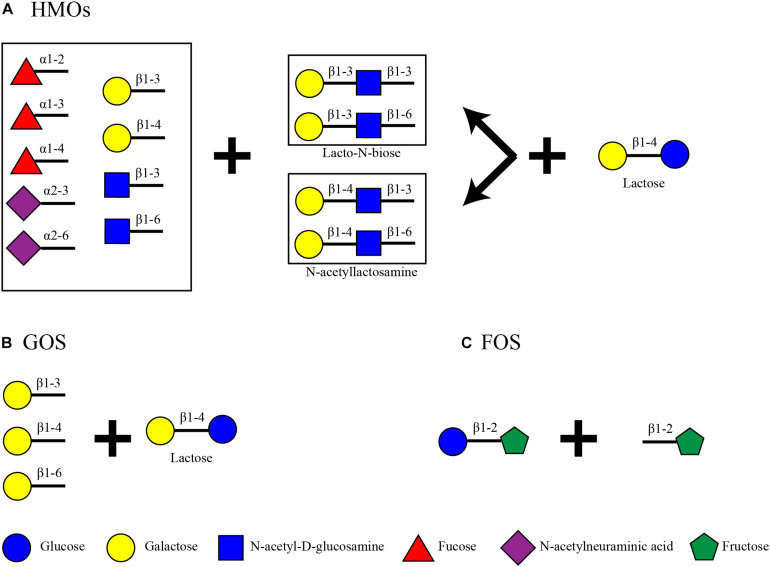
Structural backbone of **(A)** HMOs, **(B)** GOS, and **(C)** FOS.

Human milk oligosaccharides are the second most abundant carbohydrate in human milk after lactose (60 g/L) (Urashima et al., 2017). Total HMO concentrations can vary dependent on time, starting from 20 to 25 g/L in foremilk and reaching 5–20 g/L in hindmilk (Coppa et al., 1993; Thurl et al., 2010; Xu et al., 2017; Meemken and Qaim, 2018). These oligosaccharides consist of a lactose core decorated with N-acetyl-D-glucosamine (GlcNAc), D-galactose (Gal), N-acetylneuraminic acid (Neu5Ac) and L-fucose (Fuc) (Wu et al., 2010) (Figure 1). Up to date, more than 200 structures have been identified in human milk, all of which are an elongation product of 19 core structures (Urashima et al., 2017). The core structures can be categorized depending on the bond formed between galactose and N-acetyl-D-glucosamine. In lactose, Gal and glucose (Glc) are connected with β1-4 linkage creating a disaccharide. Lactose is elongated with the addition of a disaccharide with a β1-3 or β1-6 bond. These can be Lacto-N-biose, where GlcNAc is connected to Gal with a β1-3 bond, or N-acetyllactosamine, where GlcNAc is connected to Gal with a β1-4 bond. These lead to a Type I or Type II chain, respectively. The tetrasaccharide can be further decorated with Fuc or Neu5Ac or the Lacto-N-biose/N-acetyllactosamine disaccharide. The configuration of the HMO can be either linear or branched. When a disaccharide is attached to the 3N of Gal, the 6N is available and its decoration leads to a branched structure, and vice versa (Urashima et al., 2017). Up to date, there have not been any characterized structures with an additional Glc or lactose in their structure. The variability of HMOs in mothers additionally depends on their Secretor Status and Lewis blood type which is defined by the presence or absence and position of the fucose residues on the HMOs (Scheneel-Brunner et al., 1972; Viverge et al., 1990; Kelly et al., 1995; Yip et al., 2007; Underwood et al., 2015). Some HMO structures are also sialylated, thus resulting in 8–21% of the total HMO concentration (Totten et al., 2012). Fuc residues can be attached by an α1-2, α1-3, or α1-4 linkage and Neu5Ac by an α2-3 or α2-6 linkage.

On the other hand, infant formula, a common breast milk analog, does not contain HMOs. Some products, however, contain plant-based indigestible carbohydrates such as galacto-oligosaccharides (GOS) and fructo-oligosaccharides (FOS) to mimic some of the benefits of HMOs (Figure 1). GOS are made out of a lactose core elongated by Gal monomers (β1-3-Gal, β1-4-Gal, or β1-6-Gal) reaching a degree of polymerization (DP) from 2 to 8 (Verkhnyatskaya et al., 2019). FOS are made of the addition of repetitive fructose moieties to a glucose unit with a DP from 2 to 60. Dependent on the number of fructose molecules the FOS are characterized as inulin (DP = 2–60), oligofructose or long chain fructooligosaccharides (DP < 20) or short chain fructooligosaccharides (DP < 5) (Sabater-Molina et al., 2009; Akkerman et al., 2019). Inulin type FOS (Figure 1) are considered here due to their popularity as infant formula ingredients (Sorour et al., 2017).

## Glycoside Hydrolases Toward Milk Associated Oligosaccharides in Infancy: Presence and Importance

### Characterized Glycoside Hydrolases

The HMOs found in human milk are an excellent substrate for bacteria that possess the suitable enzymatic abilities to degrade them. This is suggested to stir the microbial community toward the dominance of bifidobacteria in early life gut microbiota. Research in the last decade has focused majorly *o*n the characterization of bifidobacterial (*B. breve*, *B. longum*, *B. bifidum*) enzymes to elucidate the complete degradation pattern of HMOs. Similarly, respective focus has been applied to enzymes that degrade the common prebiotics, GOS and FOS, that are added in infant formulas. Glycoside hydrolases are necessary to break the bonds that withhold the structures of these oligosaccharides. These are enzymes of hydrolytic capacity, meaning that they react with water to abolish glycosidic bonds in a retaining or inverting manner. According to their primary structure, they are classified into 167 families up to this date (Lombard et al., 2014). For this review, we have summarized the GH families that are related to the degradation of HMOs, GOS and FOS based on experimentally acquired data (Table 1). The results are restricted to enzymes that have been currently characterized by the following means in bacteria that are highly abundant in infant gut: isolation and purification, knock-out of gene and research of function, gene expression micro-arrays, proteomics or patent.

**TABLE 1:** GH families of the infant gut microbiota and their identified specific enzymes that have been found to take part in HMO, GOS and FOS degradation. Enzymes are associated with their target and the bacteria from which they have been isolated.

	GH family	Enzyme	EC number	Target	Bacteria	Gene^*a*^	References
HMO related	GH18	Endo-β-N-acetylglucosaminidase/Endoglycosidase	EC 3.2.1.96	Galβ1-3GlcNAc_2_ Galβ1-4GlcNAc_2_	*B. longum subsp. infantis* ATCC 15697	EndoBI-1	Parc et al., 2015; Karav et al., 2016; Garrido et al., 2018
					*B. longum subsp. infantis* 157F/SC142	EndoBI-2	Fukuda et al., 2011; Garrido et al., 2018
	GH20	lacto-N-biosidase	EC 3.2.1.140	GlcNAcβ1-3Gal GlcNAcβ1-6Gal	*B. bifidum* JCM1254	*lnbB*	Wada et al., 2008
		β-hexosaminidase/β-1,6-N-acetylglucosaminidase	EC 3.2.1.52		*B. bifidum* JCM1254	*BbhI, BbhII*	Miwa et al., 2010
			EC. 3.2.1.-		*B. longum subsp. longum* JCM1217	BLLJ_1391	Honda et al., 2013
					*B. longum subsp. infantis* ATCC 15697	Blon_0459, Blon_0732, Blon_2355	Garrido et al., 2012; Kavanaugh et al., 2013
	GH29	α-L-fucosidase	EC 3.2.1.51	Fucα1-3Gal Fucα1-4Gal Fucα1-3GlcNAc Fucα1-4GlcNAc	*B. longum* subsp. *infantis* ATCC 15697	Blon_0248, Blon_0426, Blon_2336	Sela et al., 2012; Kim et al., 2013
					*B. longum* subsp. *infantis* ATCC 15697	Blon_2336	Sela et al., 2012
		α-1,3/1,4-L-fucosidase	EC 3.2.1.111		*B. bifidum* JCM1254	*afcB*	Ashida et al., 2009
	GH33	2,3-2,6-a-sialidase	EC 3.2.1.18	Neu5Acα2-3Gal Neu5Acα2-6Gal Neu5Acα2-6GlcNAc	*B. longum* subsp. *infantis* ATCC15697	*nanH1, nanH2*	Sela et al., 2011
					*B. bifidum* JCM1254	*SiaBb2*	Kiyohara et al., 2011
					*B. longum* subsp. *infantis* ATCC 15697	Blon_2348	Kim et al., 2013
	GH85	Endo-β-N -acetylglucosaminidase/Endoglycosidase	EC 3.2.1.96	Galβ1-3GlcNAc_2_ Galβ1-4GlcNAc_2_	*B. longum* NCC2705 *B. longum* DJO10A *B. breve*	EndoBB	Schell et al., 2002; Garrido et al., 2018
	GH95	α-1,2-L-fucosidase	EC 3.2.1.63	Fucα1-2Gal	*B. longum* subsp. *infantis* ATCC 15697	Blon_2335	Sela et al., 2012
					*B. bifidum* JCM1254	*afcA*	Ashida et al., 2009
	GH112	GNB/LNB phosphorylase	EC 2.4.1.211	Galβ1-3GlcNAc	*B. breve* UCC2003	lnbP	James et al., 2016
					*B. bifidum* JCM1254	*LnpA1, LnpA2*	Nishimoto and Kitaoka, 2007; Nishimoto et al., 2012
	GH136	lacto-N-biosidase	EC 3.2.1.140	GlcNAcβ1-3Gal	*B. longum subsp. longum* JCM1217	*LnbX*	Sakurama et al., 2013
HMO and GOS related	GH1	β-1,4-galactosidase	EC 3.2.1.23	Galβ1-4Glc	Putative		
	GH2	β-1,4-galactosidase	EC 3.2.1.23	Galβ1-4Glc	*B. longum* subsp. *infantis* ATCC15697	*Bga2A*	Yoshida et al., 2012
					*B. longum* subsp. *infantis* ATCC 15697	Blon_2334, Blon_0268	Garrido et al., 2013; Kim et al., 2013
					*B. breve* UCC2003	*lacZ6*	James et al., 2016
					*B. breve* UCC2003	*lacZ(2)*	O’Connell Motherway et al., 2013
					*B. bifidum* DSM20215	*BIF1, BIF2, BIF3*	Møller et al., 2001
					*B. bifidum JCM1254*	*BbgIII*	Miwa et al., 2010
					**B. bifidum** NCIMB4117	*BbgI, BbgIII, BbgIV*	Goulas et al., 2009
	GH35	β-galactosidase	EC 3.2.1.23	Galβ1-4Glc	Other species		
	GH42	β-galactosidase	EC 3.2.1.23	Galβ1-4Glc Galβ1-3Gal Galβ1-4Gal Galβ1-6Gal	*B. breve* UCC2003	*galG, lntA*	James et al., 2016
					*B. breve* UCC2003	*galG, gosG*	O’Connell Motherway et al., 2013
					*B. longum* subsp. *infantis* ATCC15697	*Bga42A, Bga42B, Bga42C*	Yoshida et al., 2012; Garrido et al., 2013; Viborg et al., 2014
					*B. infantis DSM20088*	*INF1*	Møller et al., 2001
					*B. longum* subsp. *infantis* ATCC 15697	Blon_2016, Blon_2416	Kim et al., 2013
					**B. bifidum** NCIMB4117	*BbgII*	Goulas et al., 2009
GOS related	GH53	Endo-galactanase	EC 3.2.1.89	Galβ1-4Gal	*B. breve* UCC2003	*galA*	O’Connell Motherway et al., 2013
FOS related	GH32	β-fructofuranosidase	EC 3.2.1.26	Glcβ1-2Fru	*B. breve* UCC2003	*fosC*	Ryan et al., 2005
		β-fructofuranosidase/fructan β-fructosidase	EC 3.2.1.80 EC 3.2.1.26	Fruβ1-2Fru Glcβ1-2Fru	*B. longum* ATCC 15697	B.longum_l1	Ávila-Fernández et al., 2016 Warchol et al., 2002
		Exo-inulinase	EC 3.2.1.80	Fruβ1-2Fru	*B. longum* subsp. *infantis* ATCC 15697	Blon_2056, Blon_0787	Kim et al., 2013
	GH13	Sucrose phosphorylase/inulinase	EC 2.4.1.7	Glcβ1-2Fru	*B. longum* subsp. *infantis* ATCC 15697	Blon_0128, Blon_1740, Blon_0282, Blon_2453	Kim et al., 2013

*The enzymes for which the gene name is not provided are recorded by their genetic locus.

Human milk oligosaccharides are complex structures, and this trait is also depicted in the enzymatic repertoire needed to dismantle them (Table 1). When glycans are linked to peptides in the form of glycoproteins, the GH18 or GH85 endo-β-N-acetylglucosaminidases are needed to free the oligosaccharides. GlcNAc residues, termed also as sialic acid, residues are cleaved by 2,3-2,6-a-sialidases of the GH33 family. Decorated fucose, in milk of Secretor mothers, is removed via α-L-fucosidases which belong to GH29 and GH95, dependent on their specificity. In the main HMO chain, hydrolysis of the β1-3 bond in Lacto-N-biose and the β1-4 bond in N-acetyllactosamine is catalyzed by LNB/GNB phosphorylases of the GH112 family. The release of lactose from the adjacent GlcNAc is performed by lacto-N-biosidases of the GH20 and GH136 families or β-hexosaminidases/β-1,6-N-acetylglucosaminidases of GH20. The remaining lactose from HMOs as well as free human milk lactose is targeted by β-galactosidases able to hydrolyze β1-4 linkages. To date, all experimentally characterized β-galactosidases belong to the GH2 and GH42 families. There are not any characterized GH1 family β-galactosidases from highly abundant bacterial inhabitants of the infant gut. However, putative *in silico* characterized β-galactosidases from this family may prove their ability to target HMOs in the future. Accordingly, GH35 β-galactosidase activity has been described for the less abundant mucus associated bacterium *Akkermansia muciniphila*, able to catalyze the removal of Gal from the GlcNAcβ1-3Gal and GlcNAcβ1-6Gal disaccharides (Guo et al., 2018; Kostopoulos et al., 2020; Xu et al., 2020). The inherent differences in the β-galactosidases of the four families in terms of structure and substrate handling were recently explained (Kumar et al., 2019). The ability of GH2 to accumulate distinct domains and the evidence of its β-galactosidases to successfully bind lactose as well as the capacity of GH42 to actively interact with broadly linked Gal could be a possible explanation for their presence in successful utilization of HMOs.

The degradation of common infant formula oligosaccharides, GOS and FOS, requires an alternate and more concise glycoside hydrolase profile (Table 1). GOS have a less complex structure, and their degradation relies on the previously explained enzymes of the GH2 and GH42 families. The utilization of FOS requires mainly enzymes that cleave the Fuc moieties from the oligosaccharides and belong to the GH13, GH32, and GH68 families. However, agreeing to previous exploratory attempts (Chia, 2018) there were no available data for characterized GH68 enzymes in known infant gut bacteria.

### Human Milk and Alternative Feeding Enrich Pre-weaning Infants With HMO-Related GHs

**FIGURE 2:**
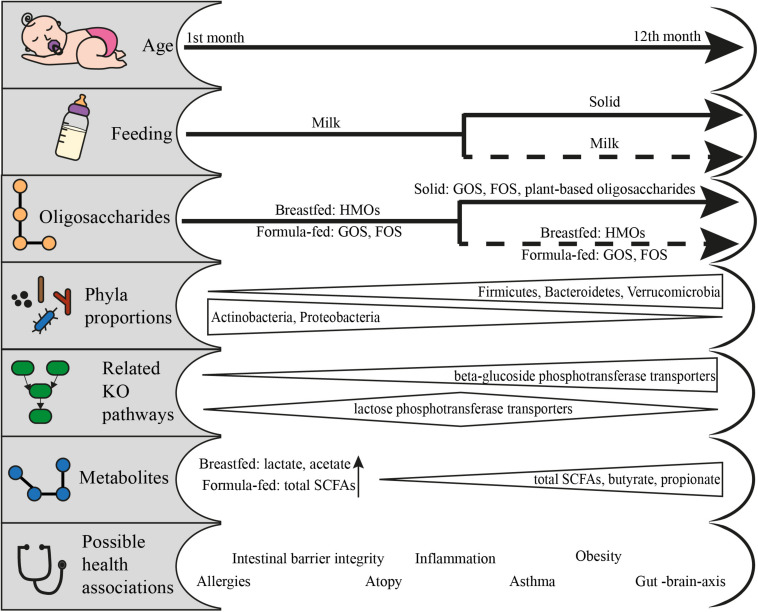
From feeding to health outcomes in the first year of life: feeding patterns and the respective available oligosaccharides, the change in phyla proportions, the KO pathways related to the utilization of fed oligosaccharides, the metabolites produced and the possible health associations. Data adapted from Schwiertz et al. (2010), Koenig et al. (2011), van der Aa et al. (2011), Bäckhed et al. (2015), Huang et al. (2017), Cait et al. (2019), Roduit et al. (2019), Venegas et al. (2019), and Differding et al. (2020).

The first diet humans come in contact with is milk. The breastfeeding lasts approximately 6 months, but depending on other factors such as the availability of the breast milk or the societal context, it can last up to 12 months or 2 years (Figure 2) (Gianni et al., 2019; World Health Organization and United Nations Children’s Fund, 2019). During that period, for many children across the globe, milk consumption also means alternative forms of feeding like infant formula. Almost 75% of infants in the western world and 60% globally will receive infant formula within the first six months (Theurich et al., 2019; World Health Organization and United Nations Children’s Fund, 2019).

Infants quickly gain microbial communities that are capable of utilizing the increased concentrations of milk carbohydrates such as lactose and HMOs (Bäckhed et al., 2015). Bäckhed et al. (2015) found that the HMO-relevant GH2, GH18, GH29, GH35, GH42, GH85, and GH95 were more enriched in breastfed compared to formula-fed children of 4 months, but in a non-significant manner. Agreeingly, Ye et al. (2019) demonstrated no significant differences in GHs based on the same cohort. These results contradict the image of differential species abundances between the two groups. The general nature of some GHs like GH2, GH13, and GH42 regarding the targeted substrate can be a factor leading to that result. It should also be taken into consideration that the enzymatic capabilities of the community have been found to be determined by its members (Bäckhed et al., 2015; Lawson et al., 2019; Ye et al., 2019). In general, bifidobacteria contain the highest amount of genetically identified CAZymes related to human milk consumption (Ye et al., 2019). This ability has been attributed to different species or even different strains of this genus within the same subject. For example, the GH29 family that includes the enzymes relevant to cleavage of fucose was only present in *Bifidobacterium infantis* strains and not *B. longum*, *B. breve* or *Bifidobacterium pseudocatenulatum* in infant metagenomes (Lawson et al., 2019). Current studies show data that justify the enzymatic contribution of more taxa that is yet to be fully elucidated (Milani et al., 2015; Turroni et al., 2016; Borewicz et al., 2020).

**FIGURE 3:**
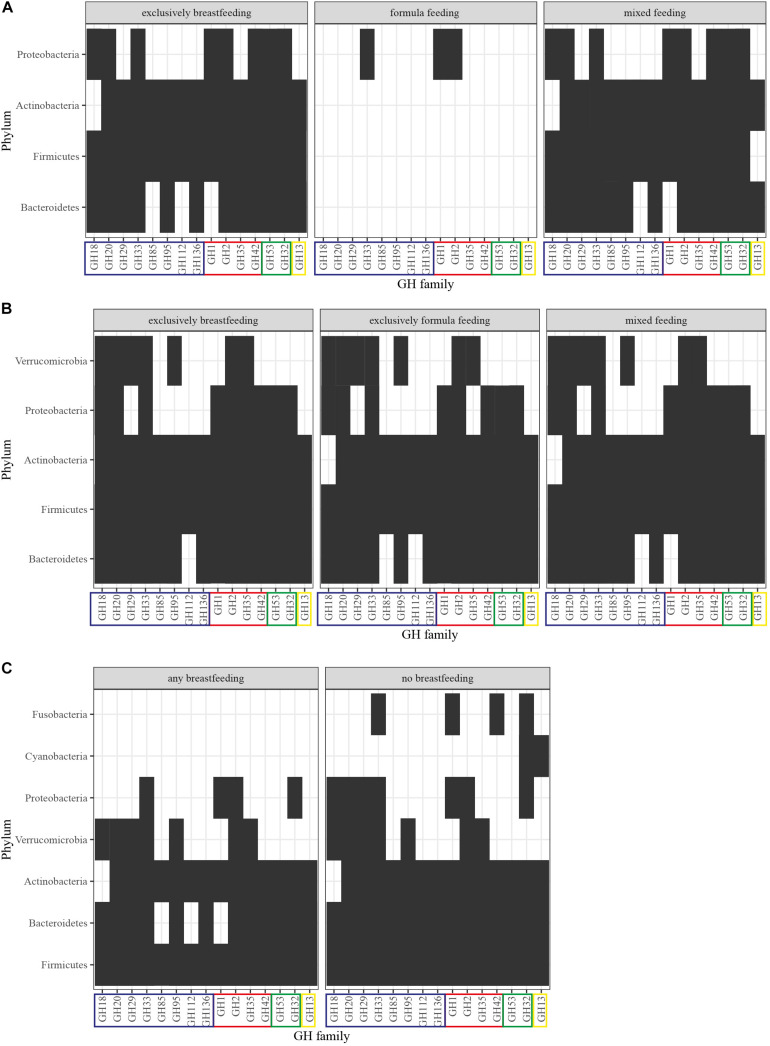
GH profiles per phylum and feeding in **(A)** newborns, **(B)** 4-month-old infants, and **(C)** 12-month-old infants. GHs were detected in MAGs of Nayfach et al. (2019) derived from the original dataset of Bäckhed et al. (2015). The identification method was based on domain-based Hidden Markov Models against the dbCAN CAZyme domain HMM database (Lombard et al., 2014; Zhang et al., 2018). GHs are grouped into: (blue) HMO-related, (red) HMO- and GOS-related, (green) GOS-related, (yellow) FOS-related. Presence of the GH family is signified with a black box and abscence with a white box.

We, therefore, proceeded to summarize the presence of HMO-, GOS- and FOS-related GHs (Supplementary Material, *In silico* Analysis Method) per phylum in the gut microbiota from infants up to 12 months of age (Bäckhed et al., 2015; Nayfach et al., 2019; Supplementary Figure 1). Our analysis illustrates that as infants reach the first 4 months of age, their gut microbiota becomes more diverse, thus contributing their GHs toward oligosaccharide utilization (Figure 3). The GH profile of formula-fed newborns is greatly depleted, which could be attributed to underrepresentation as only one out of the 98 newborns in the cohort fall within that type of feeding. At 4 months, the GHs demonstrate a coherent presence in the differently fed infants with no absent GHs. However, in children who are exclusively breastfed, Bacteroidetes, Actinobacteria and Proteobacteria contribute activity from the GH85, GH18, and GH35 families, respectively, as opposed to the exclusively formula-fed infants. These enzymatic capabilities are attributed *in silico* to: *Byturicimonas* spp., *Prevotella copri*, and *Bacteroides salyersiae* (Bacteroidetes), *Actinomyces_A neuii_A* (Actinobacteria), *Klebsiella oxytoca* and *Citrobacter* HGM20797 (Proteobacteria). Future metagenomic data from infants are needed to assess whether these traits are detected in other cohorts as well. Consistency of such results would add to the current questions on how the microbial taxa dominate the infant gut microbiota and affect physiology, initiated from feeding.

The effects of this enzymatic utilization are evident on the infants of few months old to later life. Degradation of HMOs as well as GOS and FOS leads to the production of Short Chain Fatty Acids (SCFAs), lactate and succinate. Lactate is especially dominant in the infant microbiome (Bridgman et al., 2017) and its benefits span from the cross-feeding of other bacteria (Pham et al., 2016) to the protection against pathogens (Fayol-Messaoudi et al., 2005; Duar et al., 2020) or the rejuvenation of gut epithelial cells (Lee et al., 2018). Especially breastfed infants have a higher concentration of acetate (Bridgman et al., 2017). It is interesting that Differding et al. (2020) found that the microbial communities during the milk-feeding period can get projected on the metabolome of 1-year-olds, especially for infants to which solid food is introduced early and have a more “mature” microbiota composition. Butyrate and propionate have been linked with lower occurrence of atopy coupled with experimentation on mice where SCFAs showed a promising annihilation of allergic airway inflammation (Roduit et al., 2019). The link is also evident on metagenomic data, where children whose microbiota had a lower percentage of GHs, in general as well as those related to HMO utilization, had a higher incidence of atopy (Cait et al., 2019). The same study linked those profiles with lower detection rate of genes implicated in butyrate fermentation. This agrees with the accumulated evidence on the protective nature of breastfeeding against allergy related manifestations.

### Cessation of Breastfeeding Introduces GHs From a Wider Range of Phyla

After the first six months of life, introduction of solid food becomes an important aspect of the infant diet. Breastfeeding may or may not be performed during that period. This signifies an important milestone in the composition and functionality of the gut microbiota (Figure 2). During this period, the infant microbiome has been found to mature and resemble more that of adults. The species belonging to genera such as *Bacteroides, Clostridium, Faecalibacterium, Eubacterium*, and *Ruminococcus* are introduced to the gut of children that receive solid food (Bergström et al., 2014; Bäckhed et al., 2015). However, genera dominating the younger gut such as *Bifidobacterium*, have still a pivotal role in the composition especially for the infants that continue to breastfeed (Fallani et al., 2011; Bäckhed et al., 2015). These reports further highlight the resource-pressure of human milk in shaping the gut microbiota in the first year of life.

In terms of GHs relevant to HMO, GOS, and FOS utilization (Table 1), no significant differences have been reported (Bäckhed et al., 2015; Ye et al., 2019). Interestingly, 12-month-old infants who were breastfed had a higher representation of these GHs, nevertheless in a non-significant manner (Bäckhed et al., 2015). Our previously described analysis highlights these findings and adds to the understanding of phyla contribution (Supplementary Figure 1). As seen in Figure 3, after cessation of breastfeeding, bacteria from the Fusobacteria and Cyanobacteria introduce GOS- and FOS-related GHs. However, HMO-related GHs are not depleted. Keeping in mind that GH families include various enzymes, this could be attributed to the effect of solid food on introducing a wider range of species in the gut and thus possibly increasing the overall GH genetic potential. Moreover, these enzymes are still relevant in the adult gut microbiota because they target plant- or host-derived glycans. Proteobacteria and Bacteroidetes possess HMO-related GHs (GH18, GH20, GH29 and GH85, GH112, GH1, respectively) that are absent in 1-year-olds that receive human milk as primary or side-feeding. These are *in silico* attributed to the presence of genera like *Enterobacter, Citrobacter, Hafnia*, and *Sutterella* from Proteobacteria and *Coprobacter, Butyricimonas*, and *Barnesiella* from Bacteroidetes in non-breastfed infants. Publicly available data on the coverage of reads that constitute a GH domain within a certain species could give an indication of gut microbiota composition and relationships in terms of species functional enrichment.

Bacteria that become more abundant at 1 year of age, such as *Bacteroides* spp., are known to possess variable carbohydrate degrading abilities. Moreover, evidence suggest that mucin degradation, that can be inhibited earlier by *B. longum* subsp. *infantis* (Karav et al., 2018), is performed by, for example, the increasing *Bacteroides thetaiotaomicron*, *A. muciniphila* (Bergström et al., 2014; Bäckhed et al., 2015; Differding et al., 2020) and the pre-established *B. bifidum* (Tsai et al., 1991; Derrien et al., 2004; Ruas-Madiedo et al., 2008). These *O*-glycans (Supplementary Figure 2) are targeted by some GHs that are common with the previously mentioned HMO-related CAZymes, namely fucosidases (GH29, GH95), sialidases (GH33), sulfatases, and β-hexosaminidases (GH20) (Chia, 2018; Chia et al., 2020; Kostopoulos et al., 2020). Absence of indigestible carbohydrates in gnotobiotic mice shifted the intestinal bacteria to transcriptional increase of CAZymes of mucin degradation (Desai et al., 2016), possible evidence of the protective role of prebiotics toward the integrity of the mucosal barrier. However, it has been shown that the major functional pressure of solid food is the wide range of substrates and the high amounts of plant indigestible carbohydrates. As in adults, the gut microbiota needs an array of GHs that can successfully degrade starch, pectin, xylan, arabinoxylan, arabinogalactan and other complex structures (Kaoutari et al., 2013; Bäckhed et al., 2015; Turroni et al., 2016).

Solid food affects the composition of the gut microbiota, and its results are evident in the first year and beyond. Total SCFA concentrations and the proportions of butyrate and propionate increase with solid food consumption and coincide with the proportional decrease of the non-butyrate producing bifidobacteria and the overall change in the proportion of the major phyla (Koenig et al., 2011; Differding et al., 2020). The role of microbial metabolites and the link to disease induction or protection are promising, but still quite scarce for infants, and sometimes controversial. SCFAs can have molecular interactions with intestinal cells, thus contributing to the regulation of inflammation (Venegas et al., 2019). The fortification with *Bifidobacterium*-containing symbionts protected the young intestinal cells (Zheng et al., 2020) and decreased the chances of asthma in atopic infants (van der Aa et al., 2011). SCFA-mediated effects of the gut microbiota are still emerging, with possible connection to better sleep (Szentirmai et al., 2019), protection against brain illness (Benakis et al., 2020) and behavior (Johnson and Foster, 2018). However, increased levels of SCFAs have also been correlated with obesity (Schwiertz et al., 2010; Huang et al., 2017). What is unanimously agreed, is that the first year of life is a critical timeframe for the establishment of the gut microbiota and that it has strong health implications for later life.

## Conclusion and Perspectives

In this analysis review, we have summarized the function, the importance, and the presence of GHs related to human- and formula-milk in infants up to 12 months old. We utilized publicly available metagenomic data that profile the metabolic potential of complete microbial communities. The adaptation pressure that breastfeeding imposes on those communities is evident in the contribution of phyla to these profiles. The GHs that target HMOs and plant-based formula oligosaccharides show that they both enrich the same bacterial phyla, but in a different manner with possible effects on the composition of the gut microbiota, the metabolic products, and the maturation process of the microbiome.

Such data indicate that the entire microbial network, and not only the dominant and widely characterized *Bifidobacterium* spp., contribute to the functional effect of milk-related oligosaccharides. HMO-utilizers thrive in this period and produce a variety of by-products and metabolites that are harvested by adjacent microorganisms, such as butyrate-producers. Cross-feeding is suggested to generate the interactions between the different members of the community leading to the formation of a network. This is indispensable, as it lays the ground for the mature gut microbiota. HMO-utilizers could, thus, support the first indigenous inhabitants of the adult gut microbiota. Further isolation and characterization of GHs from intestinal bacteria will contribute to the knowledge on the specificity of these enzymes, as well as the ecological advantage they confer.

Currently, the emerging genomic and analytical chemistry methods allow investigation of the composition within the gut microbiota with high resolution, as well as the quantification of its metabolic products. However, the methods capturing the actual interplay between infant gut bacteria are scarce. Moreover, the complexity of the system hinders the distinction between the effect of diet and other factors, such as the delivery mode or the surrounding environment. More metagenomics analyses are needed to unravel the potential of the infant gut microbiota to produce certain compounds and to profile the differences dependent on feeding. Currently, this second part is only based in *in vitro* experiments, leaving space for experimental procedures that link the genetic potential with the metabolic products (via meta-proteomics and meta-transcriptomics). Closed microbial systems are a suggestion for modeling the structure to function relationship that can expand the research to other species residing in the infant gut.

## Author Contributions

AI and CB initiated the ideas and concepts for this manuscript. AI wrote the manuscript. CB was involved in writing the manuscript. JK and CB supervised the project. All authors contributed to the article and approved the submitted version.

## Conflict of Interest

JK is an employee of Danone Nutricia Research. This work is part of a project partially funded by Danone Nutricia Research. The remaining authors declare that the research was conducted in the absence of any commercial or financial relationships that could be construed as a potential conflict of interest.
