# Supplementary figures and images for: Microbial Glycoside Hydrolases in the First Year of Life: An Analysis Review on Their Presence and Importance in Infant Gut
Source: Front Microbiol. 2021 May 28;12:631282. doi: 10.3389/fmicb.2021.631282 (PMC8194493; doi:10.3389/fmicb.2021.631282)

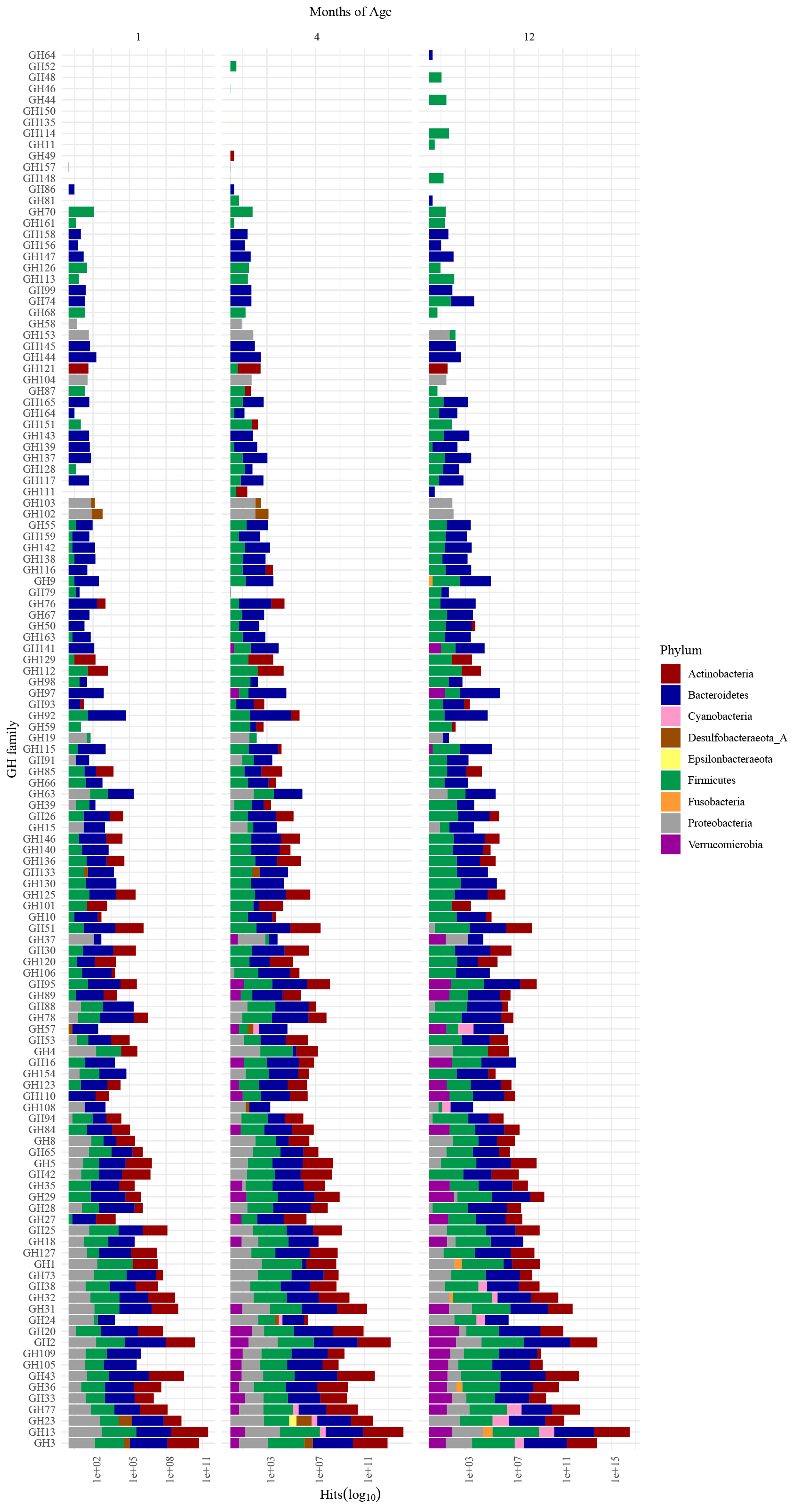

Supplement: Supplementary file 1 [file Image_1.JPEG]

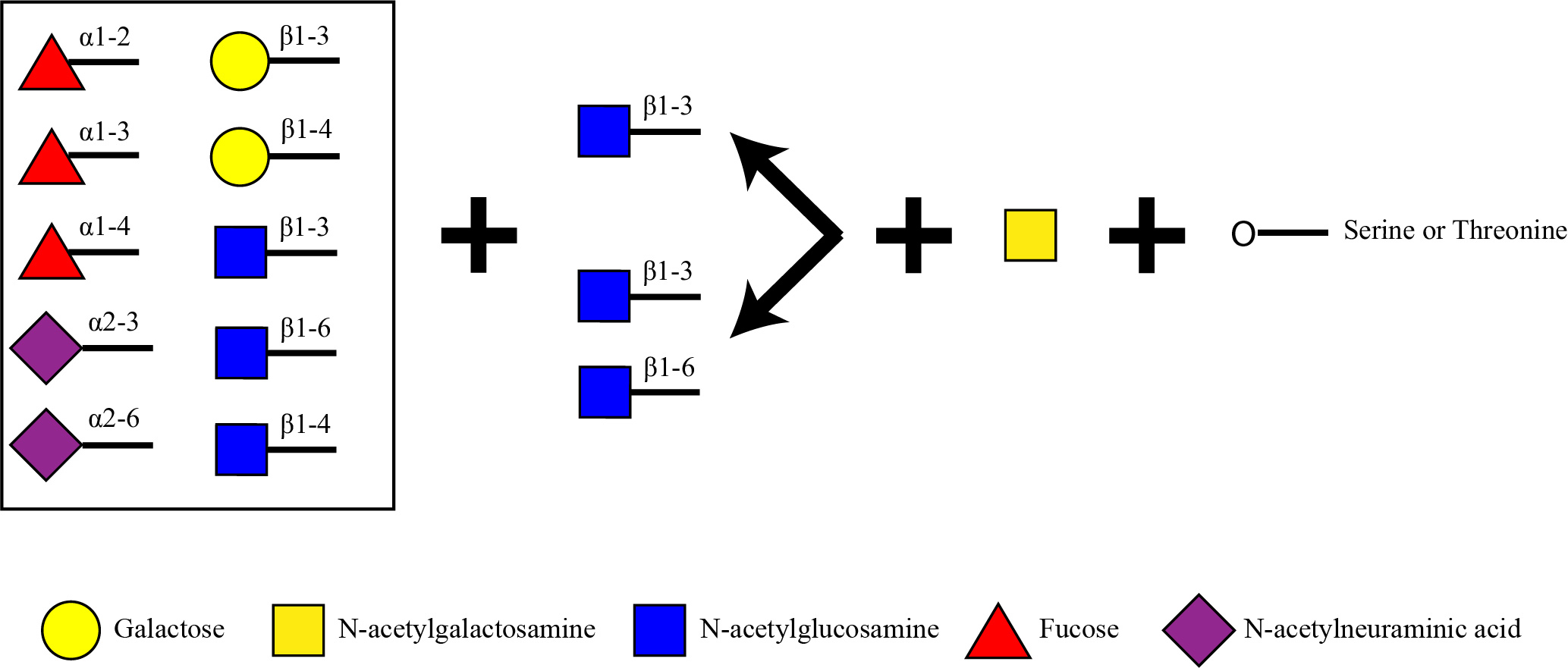

Supplement: Supplementary file 2 [file Image_2.JPEG]
