# Supplementary material for: Microbial Glycoside Hydrolases in the First Year of Life: An Analysis Review on Their Presence and Importance in Infant Gut
Source: Front Microbiol. 2021 May 28;12:631282. doi: 10.3389/fmicb.2021.631282 (PMC8194493; doi:10.3389/fmicb.2021.631282)
Supplement: Supplementary file 3 [file Table_1.docx]

Supplementary Material

# *In silico* Analysis Method

The prediction of CAZy enzymes in the MAGs of Nayfach et al. (2019) (filtered for the original data of Bäckhed et al., (2015)) was performed in our linux operating server following the instructions of the dbCAN2 meta server (Zhang et al., 2018). For the untranslated MAGs, gene prediction and translation were performed with Prodigal v2.6.3 (Hyatt et al., 2010) in normal mode. The translated assemblies were matched against the dbCAN HMM database version 8 using the -hmmscan function. The output produced by the --domtblout option was parsed with the hmmscan-parser.py tool to parse overlapping results. The output was further filtered for e-value <1e-15 and coverage >0.35, as instructed by the creators of dbCAN2 for bacterial genomes and sensitivity appropriate for prediction of glycoside hydrolases. The outputs and the available metadata were analyzed in R version 3.6.0 (R Development Core Team, 2008).

## Supplementary Figures

**Supplementary Figure 1.** Complete GH profiles per phylum in (1) newborns, (4) 4-month-old infants and (12) 12-month-old infants. The x-axis shows the log_10_ transformed number of GH domain hits in MAGs per phylum, as counted from the raw *in silico* analysis output.

**Supplementary Figure 2.** Structures of the most common forms of mucin O-glycans in the lower intestine. Information adapted from Tailford et al., (2015).

## 3. References

Bäckhed, F., Roswall, J., Peng, Y., Feng, Q., Jia, H., Kovatcheva-Datchary, P., et al. (2015). Dynamics and stabilization of the human gut microbiome during the first year of life. *Cell Host Microbe* 17, 690–703. doi:10.1016/j.chom.2015.04.004.

Hyatt, D., Chen, G. L., LoCascio, P. F., Land, M. L., Larimer, F. W., and Hauser, L. J. (2010). Prodigal: Prokaryotic gene recognition and translation initiation site identification. *BMC Bioinformatics* 11, 1–11. doi:10.1186/1471-2105-11-119.

Nayfach, S., Shi, Z. J., Seshadri, R., Pollard, K. S., and Kyrpides, N. C. (2019). New insights from uncultivated genomes of the global human gut microbiome. *Nature* 568, 505–510. doi:10.1038/s41586-019-1058-x.

R Development Core Team (2008). R: A language and environment for statistical computing.

Tailford, L. E., Crost, E. H., Kavanaugh, D., and Juge, N. (2015). Mucin glycan foraging in the human gut microbiome. *Front. Genet.* 5. doi:10.3389/fgene.2015.00081.

Zhang, H., Yohe, T., Huang, L., Entwistle, S., Wu, P., Yang, Z., et al. (2018). dbCAN2: a meta server for automated carbohydrate-active enzyme annotation. *Nucleic Acids Res.* 46, 95–101. doi:10.1093/nar/gky418.
